# Enzymatic Synthesis of Diacylglycerol-Enriched Oil by Two-Step Vacuum-Mediated Conversion of Fatty Acid Ethyl Ester and Fatty Acid From Soy Sauce By-Product Oil as Lipid-Lowering Functional Oil

**DOI:** 10.3389/fnut.2022.884829

**Published:** 2022-04-27

**Authors:** Konglong Feng, Huaiyi Fang, Guo Liu, Weijie Dai, Mingyue Song, Jiangyan Fu, Linfeng Wen, Qixin Kan, Yunjiao Chen, Yuanyou Li, Qingrong Huang, Yong Cao

**Affiliations:** ^1^Guangdong Provincial Key Laboratory of Nutraceuticals and Functional Foods, College of Food Science, South China Agricultural University, Guangzhou, China; ^2^College of Marine Sciences, Beibu Gulf University, Qinzhou, China; ^3^Guangdong Huiertai Biotechnology Co., Ltd., Guangzhou, China; ^4^Guangdong Meiweixian Flavoring Foods Co., Ltd., Zhongshan, China; ^5^Guangdong Laboratory for Lingnan Modern Agriculture, College of Marine Sciences, South China Agricultural University, Guangzhou, China; ^6^Department of Food Science, Rutgers University, New Brunswick, NJ, United States

**Keywords:** diacylglycerol, soy sauce by-product oil, enzymatic synthesis, a two-step vacuum-mediated conversion, lipid-lowering, functional oil

## Abstract

Soy sauce by-product oil (SSBO), a by-product of the soy sauce production process, is the lack of utilization due to an abundance of free fatty acid (FFA) and fatty acid ethyl ester (EE). The utilization of low-cost SSBO to produce value-added diacylglycerol (DAG)-enriched oil and its applications are promising for the sustainability of the oil industry. The objective of this study was to utilize SSBO containing a high content of EE and FFA as raw material to synthesize DAG-enriched oil and to evaluate its nutritional properties in fish. Based on different behaviors between the glycerolysis of EE and the esterification of FFA in one-pot enzymatic catalysis, a two-step vacuum-mediated conversion was developed for the maximum conversions of EE and FFA to DAG. After optimization, the maximum DAG yield (66.76%) and EE and FFA conversions (96 and 93%, respectively) were obtained under the following optimized conditions: lipase loading 3%, temperature 38°C, substrate molar ratio (glycerol/FFA and EE) 21:40, a vacuum combination of 566 mmHg within the initial 10 h and 47 mmHg from the 10th to 14th hour. Further nutritional study in fish suggested that the consumption of DAG-enriched oil was safe and served as a functional oil to lower lipid levels in serum and liver, decrease lipid accumulation and increase protein content in body and muscle tissues, and change fatty acid composition in muscle tissues. Overall, these findings were vital for the effective utilization of SSBO resources and the development of future applications for DAG-enriched oil as lipid-lowering functional oil in food.

## Introduction

High-fat intake results in an increased risk of obesity, hyperlipidemia, cardiovascular disease, and other metabolic syndromes, which is always a great concern nowadays. A novel healthy structured lipid has attracted much research interest due to its unique health benefits, such as calorie reduction, anti-obesity, and lipid-lowering properties (1). Moreover, a variety of novel structured healthy lipids are developed in recent years, in which diacylglycerol (DAG), is one of the more suitable alternatives to the conventional high-calorie triacylglycerol (TAG) oil, is a research hotspot (2). DAG consists of 1,3-DAG and 1,2-DAG forms, whose structure is constituted by a glycerol backbone esterified with two fatty acids. Previous studies have reported that the consumption of DAG can prevent weight gain and fat deposition, lowers postprandial hyperlipidemia and serum TAG levels, and improves glucose metabolism (3–9). These health benefits are ascribed to the metabolic differences of DAG oil in the compositions and positional distributions of fatty acids (10). DAG, especially 1,3-DAG, are hydrolyzed to 1(3)-MAG after digestion, which is not efficiently hydrolyzed again and reassembled as TAG. Hence, the consumption of DAG oil could not be stored up as fat in adipose tissue and the body. Nowadays, on the basics of its safety for human consumption and its many acclaimed health benefits, DAG oil is commercially available in Japan and United States (11). Nevertheless, DAG oil has not yet been a great success in the market due to non-uniform scientific reports and the lack of economic competitiveness and technical sophistication in the production of DAG (12).

**SCHEME 1:**
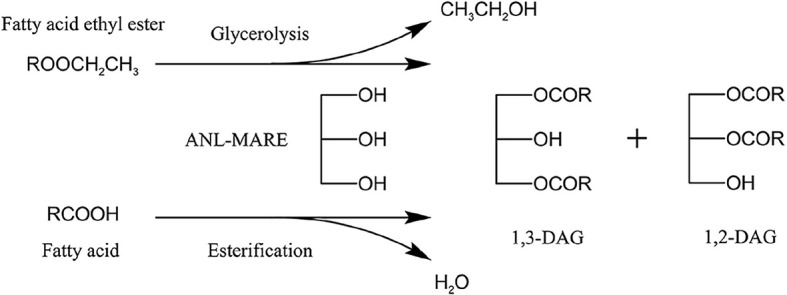
ANL-MARE catalyzed the synthesis of DAG-enriched oil by a two-step vacuum-mediated conversion of EE and FFA.

Diacylglycerol (DAG) is a minor natural component in various edible oils with a maximum level of up to 10% (13). Thus, considering the increasing demand for DAG enriched oil, many approaches have been investigated to synthesize DAG. Enzymatic synthesis of DAG was widely employed for its environmentally friendly processes, higher yield, and mild conditions (14). DAG can be produced through esterification, glycerolysis, partial hydrolysis, and so on. Among these approaches, an enzymatic esterification is a common approach for the production of DAG-enrich oil, in which fatty acids (FFA) are esterified with glycerol using lipase (15, 16). Glycerolysis is the most economical and industrial promising method for the production of DAG (17). The high content of DAG (about 60-65%) was synthesized by enzymatic glycerolysis using commercial lipases (18), and the docosahexaenoic acid-rich DAG-rich oil was also produced by lipase-catalyzed glycerolysis of microbial oil in a solvent-free system (19). Additionally, Awadallak et al. provided a new approach to producing a high content of DAG (55.6%) from linseed oil by combining enzymatic glycerolysis and esterification (20). To date, various raw materials, including triglycerides of edible oils, high-acid oil, fatty acid, and fatty acid ethyl ester (EE), have been used as a substrate for DAG production (4, 20–23). However, none of the studies have reported on the synthesis of DAG from the refined soy sauce by-product oil (SSBO) containing a high content of EE and FFA (49.96 and 30.21%, respectively). SSBO is a by-product of the soy sauce production process, and its utilization was restricted due to an abundance of free fatty acid (FFA) and dark color (24). In the previous study, an effective approach was proposed to reduce FFA content in high-acid soy sauce residue oil through low-cost and reusable biocatalysis (ANL-MARE) catalyzed esterification, which could convert high content of FFA (61.26%) to DAG-enriched soy sauce residue oil (25). Although ANL-MARE had the higher capability in the conversion of FFA to DAG, it was not effectively glycerolysis of EE in the optimum conditions for enzymatic deacidification. Besides, SSBO contains a higher content of EE in this study than that in our previous study. Thus, the optimum conditions for enzymatic deacidification were not suitable for the glycerolysis of EE. FFA and EE might be good raw materials as acyl group donors, especially for the synthesis of high content 1,3-DAG by using a 1,3 specific lipase. The higher DAG yield might be able to obtain when EE was able to effectively transform into DAG by transesterification with glycerol, along with enzymatic deacidification of SSBO. Moreover, SSBO has an advantage in large annual production and low cost. Hence, the present study proposed to use SSBO for the synthesis of valuable DAG-enriched oil by combined conversion of EE and FFA from SSBO with glycerol. Although many studies on DAG production *via* enzymatic esterification and glycerolysis have been performed, a combination of glycerolysis of EE and esterification of FFA in one-pot enzymatic catalysis is rarely studied (Scheme 1). DAG oil has unique nutritional properties concerning lower body fat metabolism (26). However, to our knowledge, little work investigates the nutritional properties of DAG-enriched oil from SSBO. It is expected that DAG-enriched oil will be used as functional oil.

In this study, DAG-enriched oil was synthesized using a low-cost SSBO containing a high content of EE and FFA as raw material and a non-commercial immobilized *Aspergillus niger* lipase (ANL-MARE) as a catalyst. Based on the different behaviors between the transesterification of EE and the esterification of FFA in one-pot enzymatic catalysis, a two-step vacuum-mediated conversion was developed for the conversions of EE and FFA to DAG. Subsequently, reaction conditions were optimized through a single factor experiment and response surface methodology (RSM). The nutritional properties of DAG-enriched oil from SSBO were evaluated in fish.

## Materials and Methods

### Materials

Food-grade glycerol and SSBO were obtained from Guangdong Huiertai Biotechnology Co., Ltd. (Guangzhou, China). SSBO was preliminary refined by desalted, degummed, dewaxed, dehydrated, and bleached, respectively. The non-commercial immobilized Aspergillus niger lipase (ANL-MARE) was prepared by Guangdong Provincial Key Laboratory of Nutraceuticals and Functional Foods (25). Edible Soybean oil was purchased from a local market. Total triglyceride (TG) and total cholesterol (TC) were supplied by Nanjing Jiancheng Bio-Engineering Institute Co., Ltd. (Nanjing, China). Chromatographic-grade n-hexane, isopropyl alcohol, and formic acid were bought from Aladdin Reagent Co. Ltd. (Shanghai, China).

### Effects of Vacuum on the One-Pot Conversion of Fatty Acid Ethyl Ester and Fatty Acid From Soy Sauce By-Product Oil

A vacuum-driven air bubbling operation was carried out in a vacuum rotary evaporator (27). Approximately 50 g of the refined SSBO, with high EE and FFA, and 3% ANL-MARE dosage were incubated for 10 min in a 250-ml round-bottom flask. Subsequently, the reaction was started by adding glycerol (molar ratio of glycerol to FFA and EE, 1:2) at 55°C under different vacuum levels with air gas bubbling. During the reaction, a 200 μl aliquot was collected from the reaction mixture at a variety of time points and analyzed by high-performance liquid chromatography (HPLC) as described below.

### A Two-Step Vacuum-Mediated Conversion of Ethyl Ester and Free Fatty Acid From Soy Sauce By-Product Oil to Diacylglycerol -Enriched Oil

According to the results in different vacuum levels, a two-step vacuum process was proposed for maximizing esterification of FFA and glycerolysis of EE. In the first step, the reaction was carried out within the initial 8 h under a low vacuum level (647 mmHg). After most of the EE was transformed, a high vacuum level (47 mmHg) was applied to transform the residual FFA in the reaction mixture from the 8th to the 10th hour.

### Optimization of Reaction Conditions for the Synthesis of Diacylglycerol

Firstly, substrate molar ratio (glycerol/FFA and EE), temperature, ANL-MARE loading, and reaction time were further optimized by a single factor experiment. The effect of a variables on the yield of DAG content, EE, and FFA conversion were analyzed.

Subsequently, response surface methodology (RSM) was used to obtain the optimal reaction conditions. Based on the results of the single factor experiment, the substrate mole ratio, temperature, substrate molar ratio (glycerol/FFA and EE), and the first step vacuum level were selected as the optimizing factors. A three-factor-three-level Box Behnken design (BBD) was used to generate factor combinations using Design Expert 8.0.6 software (Stat-Ease Inc., Minneapolis, MN, United States) (Table 1).

**TABLE 1:** Response surface methodology (RSM) design and corresponding results of diacylglycerol (DAG) yield and the conversion of EE and FFA.

Run	Temperature (°C)	GLY:EE&FFA	Vacuum (mmHg)	Final composition (%)					Conversion (%)	
				DAG	TAG	MAG	EE	FFA	EE	FFA
1	45 (0)	4:7 (1)	347 (−1)	59.80	15.59	16.01	4.78	3.81	90.43	87.39
2	35 (-1)	1:2 (0)	347 (−1)	63.00	17.40	10.34	6.14	3.13	87.72	89.66
3	45 (0)	1:2 (0)	497 (0)	62.91	25.13	7.40	1.80	2.76	96.39	90.86
4	45 (0)	4:7 (1)	647 (1)	63.26	17.39	14.37	2.27	2.72	95.46	91.01
5	35 (-1)	1:2 (0)	647 (1)	65.61	18.52	8.73	2.87	4.26	94.25	85.90
6	55 (1)	3:7 (−1)	497 (0)	50.95	39.58	3.44	3.77	2.26	92.46	92.51
7	35 (-1)	3:7 (−1)	497 (0)	61.81	19.94	4.87	5.68	7.70	88.64	74.51
8	55 (1)	1:2 (0)	647 (1)	59.74	28.79	7.36	1.94	2.18	96.12	92.80
9	55 (1)	1:2 (0)	347 (−1)	44.99	14.20	12.32	24.24	4.25	51.49	85.92
10	45 (0)	1:2 (0)	497 (0)	63.94	24.48	7.53	2.26	1.79	95.48	94.07
11	45 (0)	1:2 (0)	497 (0)	62.63	24.30	8.12	2.58	2.36	94.84	92.18
12	45 (0)	3:7 (−1)	347 (−1)	56.52	30.23	3.21	6.40	3.64	87.19	87.96
13	45 (0)	1:2 (0)	497 (0)	63.07	23.95	8.06	2.98	1.95	94.03	93.54
14	45 (0)	3:7 (−1)	647 (1)	56.74	31.68	3.35	3.81	4.42	92.37	85.37
15	55 (1)	4:7 (1)	497 (0)	47.74	13.36	18.26	14.02	6.62	71.94	78.10
16	35 (-1)	4:7 (1)	497 (0)	63.81	16.76	14.14	2.62	2.67	94.75	91.15
17	45 (0)	1:2 (0)	497 (0)	62.92	23.29	8.02	2.90	2.87	94.19	90.51

*GLY: glycerol, FFA: free fatty acid, EE: fatty acid ethyl esters, MAG: monoglyceride, DAG: diacylglycerol, TAG: triacylglycerol.*

### Analysis of Acylglycerol Composition by High-Performance Liquid Chromatography

The acylglycerol composition of raw SSBO and reaction products were determined by a normal phase HPLC (LC-15, Shimadzu, Japan) equipped with a refractive index detector. The samples were dissolved into the mobile phase (21:1:0.004, v/v/v, n-hexane/isopropyl alcohol/formic acid), and then, eluted *via* the gradient elution of the mobile phase. The chromatographic conditions were set as follows: column temperature, 30°C; flow rate, 1 mL/min; sample injection volume, 10 μl. The content of each lipid class was calculated by peak-areas percentages. The DAG content was calculated based on the sum peak area of 1,3-DAG and 1,2-DAG concerning the total peak areas. The conversions of EE or FFA were calculated as the glycerolysis of EE amount or the esterified FFA amount to the initial EE or FFA amount.

### Clinical Study, Sample Collection, and Experimental Design

Nile tilapia were supplied by Guangdong Tilapia Breeding Farm, Guangdong, China. Fish were adaptively fed for 2 weeks in the aquaculture facility of the South China Agricultural University. After acclimation, the fish were randomly divided into the DAG-enriched oil (DGO) and soybean oil (SBO) groups (150 individuals each, 6 circular tanks). The detailed components of the two experimental diets are shown in Supplementary Table 1. During the feeding experiment, the fish were fed the diets twice a day. At the end of 8 weeks trial, all fish from each tank were weighed and measured individually before being anesthetized with clove oil. Twelve fishes per group (4 fish/tank) were used for physiological and biochemical analyses. Plasma samples were obtained from blood by centrifugation for further serum biochemical analysis. Once the blood sampling was finished, the liver and adipose tissue were individually collected and weighed after sacrifice. The muscle was dissected without skin. A part of muscle tissue was used for texture analysis and another part was frozen for further analysis. All samples were rapidly frozen in liquid nitrogen and then stored at –80°C.

### Biochemical Estimation of Serum and Liver

The serum TC, TG, high-density lipoprotein cholesterol (HDL-C), low-density lipoprotein cholesterol (LDL-C), alanine transaminase (ALT), and aspartate transaminase (AST) levels were determined using an automatic biochemical analyzer (Beckman Coulter chemistry analyzer AU5800 series, Tokyo, Japan), according to manufacturer’s instruction. The hepatic TG and TC levels were measured using commercially available kits (Nanjing Jiancheng Bioengineering Institute, Nanjing, China).

### Analysis of Fatty Acids Composition in Muscle by Gas Chromatography-Mass Spectrometry

The determination of fatty acid composition in muscle was performed according to a previously described method with some modifications (28). The muscle samples were dried by vacuum freeze-drying, and then, the total lipids of the liver were extracted from the dried muscle samples according to Folch’s procedure (29). Fatty acid methyl esters were prepared by referring to the method of Liu et al. (30) and then determined using GC-MS (Agilent 6890-5973N, Agilent Technologies, United States) equipped with a DB-WAX capillary column (60 m, 0.25 μm i.d., film thickness, Agilent Technologies, United States). The results were presented as the percentage of each fatty acid concerning the total fatty acids.

### Analysis of the Proximate Composition of Whole Fish and Muscle Tissues

Six fish or 6 muscle samples were randomly selected from each group for measuring the proximate composition of whole fish and muscle tissues. The determination of proximate composition, including the contents of moisture, ash, crude lipid, and crude protein, was performed according to a previously described method (31).

### Characterization of Muscle Texture Properties and Edible Quality

Six fresh muscle samples per group were used for measurement of muscle texture properties and edible quality after the muscle was dissected without skin. The muscle texture properties were assayed according to the method of Wang et al. (32). The edible quality was assayed according to the method of Lv et al. (31).

### Statistical Analysis

Data were expressed as means ± SD. The data in the enzymatic synthesis of DAG were analyzed by using a one-way analysis of variance (ANOVA) with SPSS 21.0 software (IBM Corporation, Armonk, New York), and different letters were indicated significant differences according to Duncan s multiple range test (*P* < 0.05). Additionally, differences between the SBO and DGO groups were determined with Student’s *t*-test. *P* < 0.05 was considered statistical significance.

## Results

### A Two-Step Vacuum-Mediated Conversion of Ethyl Ester and Free Fatty Acid From Soy Sauce By-Product Oil to Diacylglycerol-Enriched Oil

**FIGURE 1:**
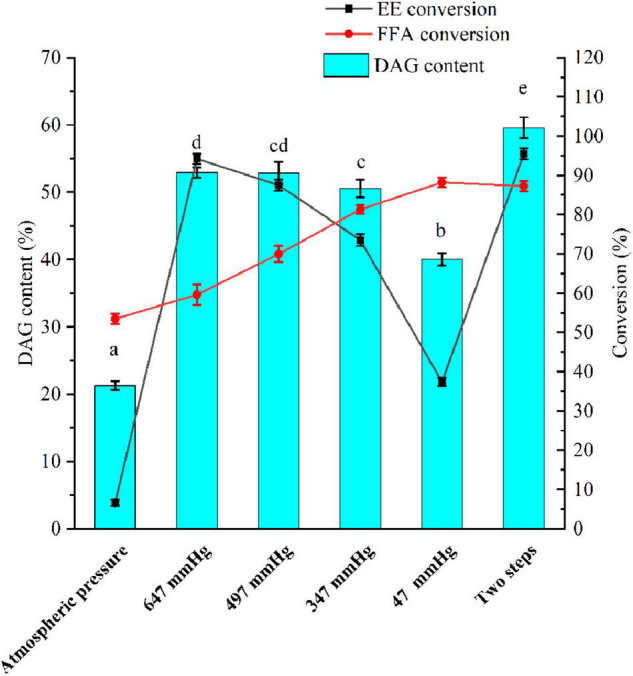
A two-step vacuum-mediated conversion of fatty acid ethyl ester and fatty acid from soy sauce by-product oil (SSBO) to diacylglycerol (DAG)-enriched oil. Reaction condition: SSBO with high ethyl esther (EE) and free fatty acid (FFA): 50 g, ANL-MARE load: 3%, substrate molar ratio (glycerol/EE and FFA): 1:2, temperature: 55°C, different vacuum with air bubbling or two-step vacuum process, 150 rpm and reaction time: 8 h or 10 h. FFA: free fatty acid, EE: fatty acid ethyl esters, DAG: diacylglycerol. The different letters indicate significant differences at *P* < 0.05.

**FIGURE 2:**
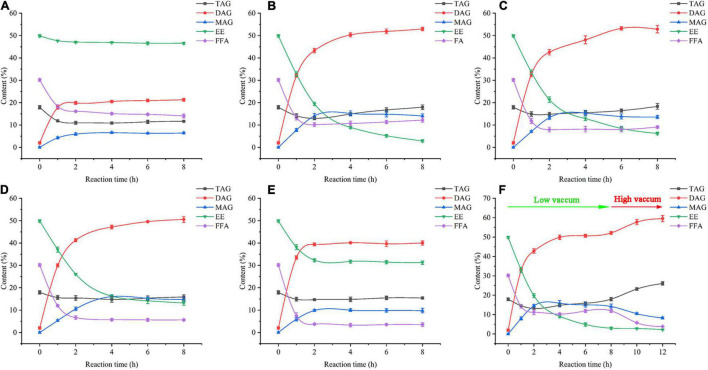
Time course of lipid composition analysis under the condition of various vacuum **(A)** Atmospheric pressure, **(B)** 647 mmHg, **(C)** 497 mmHg, **(D)** 347 mmHg, **(E)** 47 mmHg, and **(F)** Two-step vacuum process). FFA: free fatty acid, EE: fatty acid ethyl esters, MAG: monoglyceride, DAG: diacylglycerol, TAG: triacylglycerol.

A vacuum was employed to remove produced water and ethanol in a vacuum-driven air bubbling operation, thereby increasing the conversion of FFA and EE, as well as the yield of DAG. The EE and FFA from SSBO were served as acyl group donors in this reaction (Scheme 1), while the lipase showed different behaviors between the glycerolysis of EE and the esterification of FFA with glycerol (Figures 1, 2). Under atmospheric pressure, the conversion of FFA achieved about 50% during the first 2 h of the reaction, while only a little EE was transformed (Figure 2A). Also, no significant changes in the conversion of FFA and EE were observed after 2 h. As the vacuum levels increased from 647 to 47 mmHg, the conversion of FFA gradually enhanced the maximum (88.24%). The conversion of EE significantly increased as the vacuum levels increased from atmospheric pressure to 647 mmHg, up to the maximum value (94.24%), but decreased significantly as the vacuum levels increased above 647 mmHg. Meanwhile, the yield of DAG showed a similar tendency, achieving a maximum (52.93%) at 647 mmHg (Figure 1). From these results, it is clear that a high vacuum level favored the esterification of FFA, whereas a low vacuum level was conducive to the glycerolysis of EE. Vacuum levels higher than 647 mmHg were not suitable for glycerolysis of EE but accelerated the esterification of FFA, which might be attributed to transesterification activity drop with lower water content and the shifted equilibrium to esterification in lower water content. The water content or water activity of the system was likely associated with a vacuum. These results also implied that the water activity requirements for optimum efficacy were higher for glycerolysis of EE than that for esterification of FFA. Although the glycerolysis of EE and the esterification of FFA happened at the same time, EE and FFA did not convert maximally under a vacuum level. Thus, to maximize the synthesis of DAG, a two-step vacuum process was proposed for maximizing the esterification of FFA and glycerolysis of EE (Figure 2F). In the first step, the reaction was carried out within the initial 8 h under a low vacuum level (647 mmHg), in which the transesterification activity of EE was superior to the esterification activity of FFA. After most of the EE was transformed, a high vacuum level (47 mmHg) was applied to transform the residual FFA in the reaction mixture. In this approach, the conversions of EE and FFA were 95.46 and 87.28%, respectively. And this approach exhibited a conversion to DAG as high as 59.57%, which was notably higher than the single vacuum process. Therefore, a two-step vacuum process was used for the subsequent experimentation.

### Optimization of Reaction Conditions for the Synthesis of Diacylglycerol Using Single Factor Experiment and Response Surface Methodology

#### Effects of Substrate Molar Ratio (Glycerol/Free Fatty Acid and Ethyl Ester)

**FIGURE 3:**
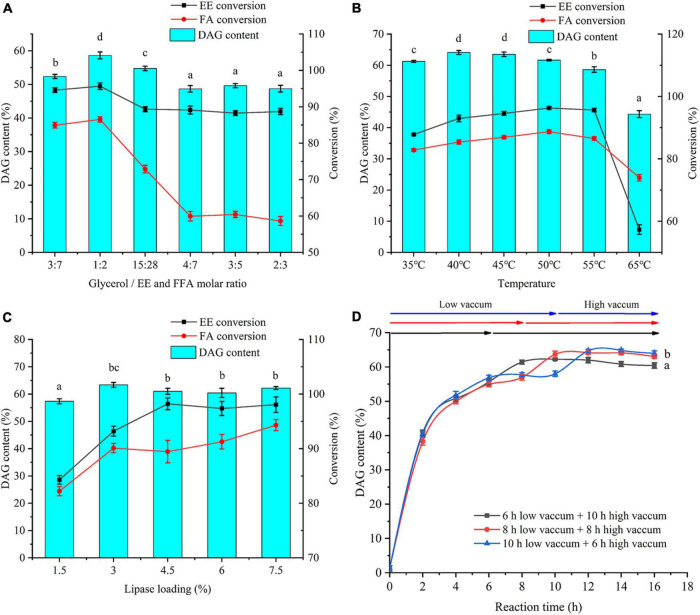
Effects of substrate molar ratio (glycerol/FFA and EE) **(A)**, temperature **(B)**, ANL-MARE loading **(C)**, and reaction time **(D)** on DAG content, EE, and FFA conversion. The different letters indicate significant differences at *P* < 0.05.

To maximize the synthesis of DAG, reaction conditions, including substrate molar ratio, temperature, ANL-MARE loading, and reaction time, were further optimized. As a substrate in the reaction, glycerol not only shows poor miscibility in the reaction system that decreases the reaction rate but also exhibits a formidable water binder that decreases water activity in the reaction system (21, 33). Hence, the optimization of the substrate molar ratio of the glycerol to FFA and EE was crucial to the product composition and the performance of immobilized lipase. The molar ratio of glycerol to FFA and EE exhibited an apparent effect on the product composition (Supplementary Figure 1A). The DAG yield markedly increased with the increase of molar ratio from 3:7 to 1:2, but remarkably declined and then remained unchanged when the molar ratio was increased greater than 1:2 (Figure 3A). The conversion of EE and FFA showed a similar tendency. Although increasing glycerol amount could enhance DAG yield to some extent, excess presence of glycerol not only decreased reaction rate, but also had a negative effect on the stability of immobilized lipase (34). Therefore, a 1:2 molar ratio was the most appropriate substrate molar ratio for further optimization.

#### Effects of Temperature

Temperatures ranging from 35 to 65°C were optimized (Figure 3B). Reaction temperature had a significant effect on the product composition (Supplementary Figure 1B). The highest DAG content was achieved as 64.11% at 40°C. As the temperature further increased from 45 to 55°C, the DAG yield decreased slowly, but the content sharply declined at 65°C. Conversely, the content of TAG showed a continuously increasing tendency with temperature increasing from 35 to 55°C (Supplementary Figure 1B). The findings were directly in line with previous findings, in which higher temperatures promote acyl migration and are conducive to synthesizing TAG (35). Although the highest DAG content was obtained at 40°C, the conversions of EE and FFA were lower at 40°C than those at 45°C. Thus, to obtain the highest conversion to DAG and the higher conversions of EE and FFA, a temperature of 45°C was chosen for further experimentation.

#### Effects of ANL-MARE Loading

As shown in Figure 3C, the DAG yield significantly increased with the increasing lipase loading from 1.5% to 3% and remained unchanged after further increased lipase loading over 3%. The highest DAG content was obtained as 63.37% at ANL-MARE loading of 3%. As the lipase loading increased, the contents of FFA and EE continuously declined, while the TAG content continuously increased (Supplementary Figure 1C). This could be ascribed to the catalytic efficiency being significantly enhanced with more addition of lipase. Therefore, considering the economy, the ANL-MARE loading of 3% was selected in this study.

#### Effects of Reaction Time

In this study, three-time processes were optimized (Figure 3D). The DAG content elevated rapidly within the initial 2 h and raised slowly after 8 h. A significant increase in the conversion of EE was observed within the time under a low vacuum, whereas the increment was insignificant after the high vacuum was applied (Supplementary Figure 2A). The maximum conversion of EE was observed at the time process, in which a high vacuum in the second step was applied after 10 h. The conversion of FFA increased rapidly within the initial 2 h, achieving about 68% (Supplementary Figure 2B). Then, it somewhat decreased due to the accumulation of produced water under low vacuum, but significantly increased until high vacuum was applied. Therefore, to maximize the conversions of EE and FFA, the reaction was used low vacuum (647 mmHg) within the initial 10 h, and then, high vacuum from the 10th to 14th hour in this study. Under this time process, 64% DAG was obtained and the conversion of EE and FFA were achieved at 96.42 and 95.34%, respectively.

#### Analysis of Response Surface Methodology for the Optimum Conditions

**FIGURE 4:**
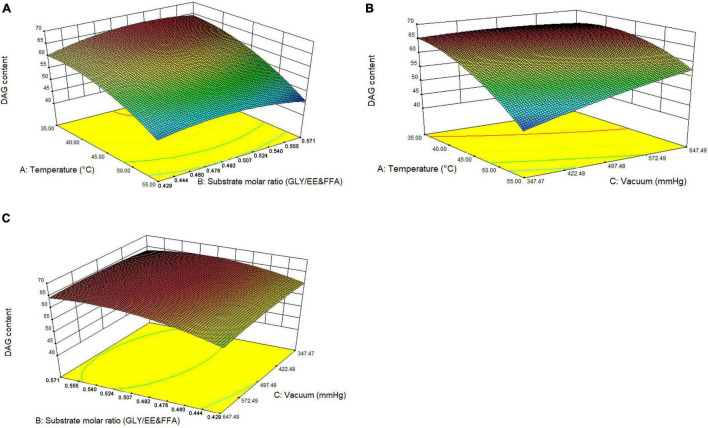
3D response surface graphs for the pairwise interactive effects on the conversion of EE and FFA to DAG yield. **(A)** temperature *vs.* substrate molar ratio, **(B)** temperature *vs.* vacuum, **(C)** substrate molar ratio *vs*. vacuum.

To further investigate the relationship and the interaction between any two parameters on DAG yield, a three-level three-factor BBD was employed (Table 1). As shown in Table 2, the model was significant at *P* < 0.001, and the multiple correlation coefficient of *R*^2^ and the adjusted *R*^2^ were 0.9311 and 0.8426, respectively. These results from ANOVA suggested that the models were suitable for predicting the DAG yield within the range of variables determined. According to the significance of each coefficient in Table 2, the independent factors, temperature, and the initial vacuum, the quadratic terms of temperature and substrate molar ratio, and the interactions between temperature and initial vacuum had significant influences on the DAG yield. The results implied that variables of temperature and initial vacuum were acted a critical role in the DAG yield and showed a negative linear relation to the DAG yield. An increment of temperature and vacuum resulted in a reduction of DAG yield. The relationships between independent variables and DAG yield in 3D response surfaces were shown in Figure 4. Based on the analysis of RSM, the optimal conditions for maximum DAG yield (66.13%) were a temperature of 37.96°C, a substrate molar ration of 211:400, and the first step vacuum level of 566 mmHg. Under the predicted optimal conditions, we slightly modified conditions for experimental feasibility and convenience. The modified conditions were as follows: a temperature of 38°C, a substrate molar ration of 21:40, the first step vacuum level of 566 mmHg within the initial 10 h, and the second step vacuum level of 47 mmHg from 10th to 14th hour. Under the modified optimal conditions, a yield of 66.39% of DAG was obtained experimentally, which showed a good agreement with the predicted values and further proved the validity of models.

**TABLE 2:** Regression coefficients and analysis of variance for response surface model fitting.

Source	Sum of squares	*df*	Mean square	*F*-value	*P*-value	Significant ^1^
Model	550.56	9.00	61.17	10.52	0.0026	significant
A-Temperature	322.61	1.00	322.61	55.46	0.0001	**
B-Substrate molar ratio	9.22	1.00	9.22	1.58	0.2485	
C-Vacuum	55.30	1.00	55.30	9.51	0.0177	*
AB	6.78	1.00	6.78	1.16	0.3162	
AC	36.80	1.00	36.80	6.33	0.0401	*
BC	2.64	1.00	2.64	0.45	0.5223	
A^2^	63.37	1.00	63.37	10.89	0.0131	*
B^2^	41.35	1.00	41.35	7.11	0.0322	*
C^2^	3.24	1.00	3.24	0.56	0.4795	
Residual	40.72	7.00	5.82			
Lack of Fit	39.73	3.00	13.24	53.36	0.0011	significant
Pure Error	0.99	4.00	0.25			
Cor Total	591.28	16.00				
*R* ^2^	0.9311					
Adj *R*^2^	0.8426					

^1^significant difference was indicated by * P < 0.05 and ** P < 0.01, respectively.

### Scale-Up Trial and Physicochemical and Compositional Characterization of Diacylglycerol-Enriched Oil

To further prepare DAG-enriched oil for nutritional studies, a 20-time substrate (1 kg high-acid oil) scale-up trial was performed under the above-optimized process conditions. After 14 h enzymatic reaction, DAG content in the crude product was approximately 66%, suggesting that the reaction was scalable.

Physicochemical properties and composition of the preliminary refined SSBO with high EE and FFA and DAG-enriched oil were determined (Table 3). After catalytic reaction, DAG-enriched oil contained a higher content of DAG, achieving 66.76% (62.62% of 1,3-DAG and 4.14% of 1,2-DAG), followed by 19.31% TAG, 10.04% monoglyceride (MAG), 1.88% EE, and 2% FFA. In addition, DAG-enriched oil also showed a low acid value (3.79 mg KOH/g) and peroxide value (3.28 mmol/kg), which reached the food additive standard of mono and diglycerides. The fatty acid composition of DAG-enriched oil was mainly composed of 48.07% linoleic acid, 23.71% oleic acid, 12.80% palmitic acid, 8.99% linolenic acid, and 5.02% stearic acid, which was similar to soybean oil. Meanwhile, the energy value of DAG-enriched oil (39.16 kJ/g) was also similar to that of soybean oil (39.53 kJ/g). Although the fatty acid composition and energy value of DAG-enriched oil resembled those of soybean oil, DAG-enriched oil contained a higher content of DAG than soybean oil (0.92%). Consumption of oils with higher content of DAG, particularly 1,3-DAG, has positive physiological effects in anti-obesity and the reduction of postprandial TAG levels (26). In this study, DAG-enriched oil had a high content of DAG (66.76%), especially a high content of 1,3-DAG, after enzymatic reaction, which might show a higher nutritive value than that of TAG oil. Hence, further studies need to investigate the nutritional properties and applications of this DAG-enriched oil.

**TABLE 3:** Physicochemical and compositional characterization of DAG-enriched oil.

	Soybean oil (SBO)	Refined SSBO	DAG-enriched oil (DGO)
Acid value (mg/g)	0.64 ± 0.03	57.73 ± 0.39	3.79 ± 0.24
Peroxide value (mmol/kg)	3.88 ± 0.47	3.05 ± 0.28	3.28 ± 0.23
Energy value (kJ/g)	39.53 ± 0.11	-	39.16 ± 0.09
Lipid content (%)			
TAG	98.64 ± 0.27	18.33 ± 0.69	19.31 ± 0.76
DAG	0.92 ± 0.13	1.49 ± 0.46	66.76 ± 1.37 (62.62% 1,3-DAG, 4.14% 1,2-DAG)
MAG	0.00 ± 0.00	0.00 ± 0.00	10.04 ± 0.42
FFA	0.44 ± 0.09	30.21 ± 0.89	2.00 ± 0.38
EE	0.00 ± 0.00	49.96 ± 0.62	1.88 ± 0.25
Fatty acid composition			
C14:0	0.11 ± 0.00	0.11 ± 0.01	0.08 ± 0.02
C16:0	12.78 ± 0.07	13.59 ± 0.26	12.80 ± 0.10
C16:1	0.15 ± 0.00	0.16 ± 0.04	0.08 ± 0.00
C18:0	5.35 ± 0.09	5.63 ± 0.38	5.02 ± 0.02
C18:1	26.65 ± 0.16	24.07 ± 0.77	23.71 ± 0.04
C18:2	46.57 ± 0.31	45.97 ± 1.74	48.07 ± 0.80
C18:3	6.99 ± 0.15	9.15 ± 0.33	8.99 ± 0.02
C20:0	0.50 ± 0.01	0.46 ± 0.02	0.41 ± 0.00
C20:1	0.31 ± 0.07	0.28 ± 0.02	0.31 ± 0.00
C22:0	0.59 ± 0.03	0.59 ± 0.03	0.52 ± 0.02

*GLY: glycerol, FFA: free fatty acid, EE: fatty acid ethyl esters, MAG: monoglyceride, DAG: diacylglycerol, TAG: triacylglycerol, SBO: soybean oil, SSBO: soy sauce by-product oil, DGO: DAG-enriched oil.*

### Nutritional Studies of Diacylglycerol-Enrich Oil in Fish

#### Effect of Dietary Diacylglycerol-Enriched Oil on Growth Performance of Fish

To investigate the nutritional effect of dietary DAG-enriched oil, we used fish (Nile tilapia) as a model organism. Soybean oil, a common edible oil, was chosen as reference TAG oil, for whose fatty acid composition was similar to that of DAG-enriched oil. In this study, the fish were fed a diet containing 4.3 wt% fat in the form of DAG-enriched oil or soybean oil for 8 weeks. As shown in Table 4, there were no significant differences in growth performance, including final body weight, weight gain, specific growth rate, feed intake, survival rate and condition factor, between the SBO and DGO groups (*P* > 0.05). Moreover, no significant changes in hepatosomatic index and intraperitoneal fat ratio were observed between the SBO and DGO groups (*P* > 0.05). Notably, viscerosomatic index significantly decreased in the DGO group compared with the SBO group (*P* < 0.05). Overall, dietary DAG-enriched oil did not affect the growth performance of fish. The results also implied that the DAG-enriched oil was proven to be safe.

**TABLE 4:** Effects of DAG-enriched oil on growth performance, feed coefficient, and organ indices in fish.

	SBO group	DGO group
Initial body weight (g)	16.91 ± 0.23	16.93 ± 0.27
Final body weight (g)	124.28 ± 6.06	121.15 ± 8.33
Weight gain rate (%)	594.73 ± 26.92	565.51 ± 35.11
Specific growth rate (%)	3.46 ± 0.07	3.38 ± 0.09
Feed coefficient	1.12 ± 0.08	1.23 ± 0.07
Feed intake (g)	2816.33 ± 56.42	2925.70 ± 73.06
Survival rate (%)	94.67 ± 6.11	93.33 ± 8.33
Condition factor (g/cm^3^)	3.41 ± 0.13	3.47 ± 0.10
Viscerosomatic index (%)	9.09 ± 0.92	8.50 ± 0.65*
Hepatosomatic index (%)	1.53 ± 0.33	1.56 ± 0.12
Intraperitoneal fat ratio (%)	0.64 ± 0.03	0.60 ± 0.04

* indicates significant differences between the SBO and DGO groups at P < 0.05.

#### Effect of Dietary Diacylglycerol-Enriched Oil on Lipid Levels in Serum and Liver

**FIGURE 5:**
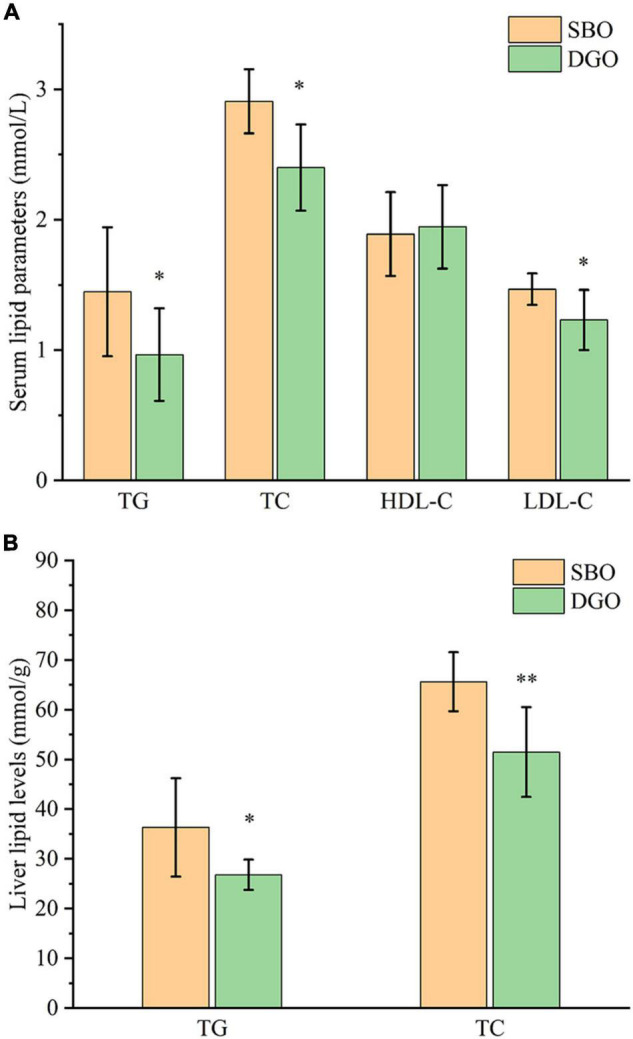
Effects of DAG-enriched oil on serum **(A)** and liver **(B)** lipid levels in fish. * and ^**^ indicate significant differences between the SBO and DGO groups at *P* < 0.05 and *P* < 0.01, respectively. TG: total triglyceride, TC: total cholesterol, HDL-C: high-density lipoprotein cholesterol, LDL-C: low-density lipoprotein cholesterol, SBO: soybean oil, DGO: DAG-enriched oil.

Compared with fish fed an SBO diet, fish fed a DAG-enriched oil diet had significantly lower serum levels of TG, TC, and LDL-C by 33%, 17%, and 16%, respectively (*P* < 0.05), but did not affect serum HDL-C, ALT, and AST levels (Figure 5A and Supplementary Figure 5). Furthermore, dietary DAG-enriched oil remarkably decreased hepatic TG and TC levels by 26% and 21%, respectively (*P* < 0.05) (Figure 5B). The results indicated that dietary DAG-enriched oil could lower lipid levels in serum and liver.

#### Effect of Dietary Diacylglycerol-Enriched Oil on Proximate Composition of Whole Fish and Muscle Tissues

**FIGURE 6:**
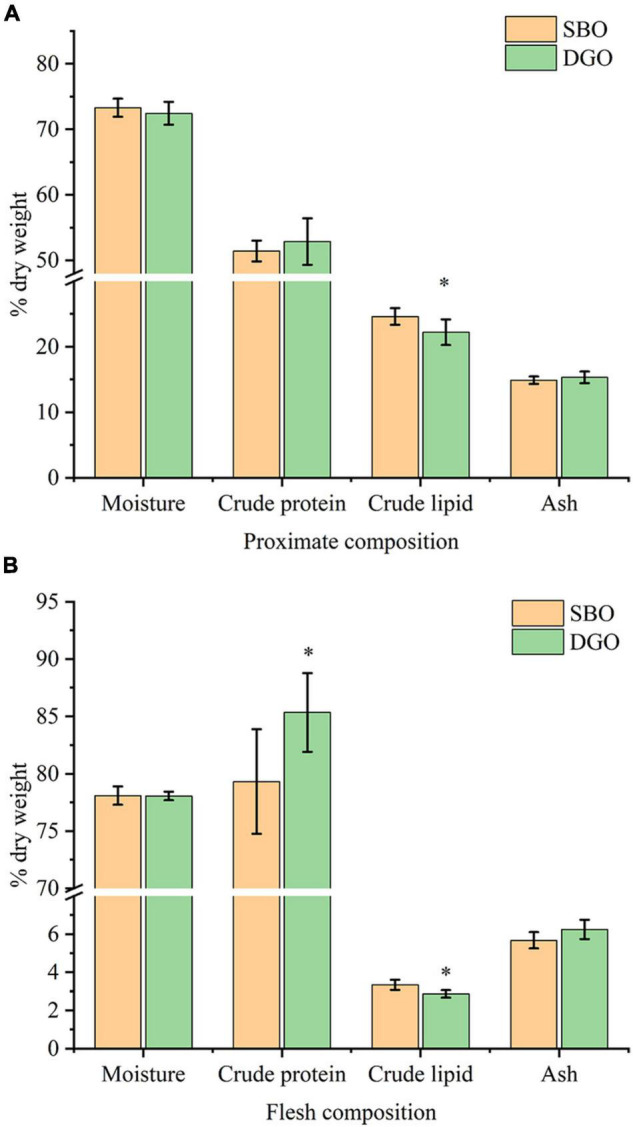
Effects of DAG-enriched oil (DGO) on the nutrient composition of whole-body **(A)** and muscle tissues **(B)** in fish. * indicates significant differences between the SBO and DGO groups at *P* < 0.05.

Compared with the SBO group, crude lipid content in the whole body was significantly lowered in the DGO group, and crude protein content in whole body was slightly increased but not significantly different (Figure 6A). Moreover, dietary DAG-enriched oil reduced significantly crude lipid content in muscle and increased crude protein content (Figure 6B). The contents of moisture and ash in the whole body and muscle were barely different between the SBO and DGO groups as shown in Figure 6. Therefore, dietary DAG-enriched oil could decrease lipid accumulation, and increased protein content in the whole fish and muscle tissues.

#### Effect of Dietary Diacylglycerol-Enriched Oil on Textural Properties and Edible Quality of Muscle Tissues

The textural properties and edible quality of fish muscle are shown in Table 5. All textural properties of muscle were similar in fish from SBO and DGO groups (*P* > 0.05). Dietary DAG-enriched oil had no changes on cooking percentage but had significantly a lower water loss rate than that of consumption of SBO, suggesting that dietary DAG-enriched oil could improve edible quality of muscle tissues.

**TABLE 5:** Effects of DAG-enriched oil on textural properties and edible quality in fish.

Muscle quality	SBO group	DGO group
Textural properties		
Hardness (gf)	339.65 ± 54.15	336.93 ± 51.94
Viscosity (mJ)	−2.75 ± 0.76	−2.75 ± 1.12
Springiness (mm)	0.26 ± 0.04	0.26 ± 0.04
Chewiness (mJ)	26.96 ± 7.38	27.36 ± 8.78
Gumminess (mJ)	99.97 ± 15.24	99.95 ± 18.17
Cohesiveness	0.29 ± 0.03	0.30 ± 0.04
Resilience	0.35 ± 0.01	0.35 ± 0.03
Edible quality		
Cooking percentage (%)	78.37 ± 1.61	79.98 ± 4.62
Water loss rate (%)	5.52 ± 1.04	3.92 ± 1.11*

*indicates significant differences between the SBO and DGO groups at P < 0.05.

#### Effect of Dietary Diacylglycerol-Enriched Oil on Fatty Acid Composition of Muscle Tissues

Overall, dietary DAG-enriched oil showed no changes on the content of total saturated fatty acids (SFA), monounsaturated fatty acids (MUFA) and polyunsaturated fatty acids (PUFA) (Table 6). Notably, for a specific FA, C18:3n3, C20:2n6, and C20:3n3 contents in muscle were significantly increased in fish fed a DAG-enriched oil diet compared to consumption of SBO, while C18:2n6 content was significantly lowered (*P* < 0.05). In addition, a lower content of n-6 PUFA and a higher ratio of n-3/n-6 PUFA were observed in a fish-fed DAG-enriched oil diet compared to consumption of SBO. The content of n-3 PUFA in the DGO group tended to be higher than that in the SBO group but the difference was not significant (*P* > 0.05). The results indicated that dietary DAG-enriched oil could change fatty acid composition in muscle tissues.

**TABLE 6:** Effects of DAG-enriched oil on muscle fatty acid composition of fish.

Fatty acids	SBO group	DGO group
C14:0	1.83 ± 0.52	2.69 ± 0.79
C16:0	22.17 ± 2.47	18.83 ± 2.52*
C18:0	11.13 ± 0.51	11.45 ± 1.62
C20:0	0.33 ± 0.37	0.39 ± 0.09
ΣSFA^1^	35.46 ± 2.00	33.36 ± 2.29
C16:1	1.92 ± 1.02	3.26 ± 1.67
C18:1n9	23.08 ± 4.63	23.76 ± 1.81
C20:1	0.89 ± 0.77	1.69 ± 0.52
C22:1n9	0.47 ± 0.41	0.41 ± 0.22
ΣMUFA^2^	26.36 ± 4.91	29.13 ± 0.88
C18:2n6	23.44 ± 1.41	20.60 ± 2.19*
C18:3n6	1.00 ± 0.08	1.12 ± 0.22
C18:3n3	1.61 ± 0.80	3.32 ± 0.54**
C20:2n6	1.41 ± 0.72	2.27 ± 0.52*
C20:3n3	1.99 ± 0.25	2.72 ± 0.50*
C20:4n6	3.60 ± 1.07	2.70 ± 0.27
C20:4n3	1.20 ± 0.65	1.47 ± 0.25
C20:5n3	2.54 ± 1.20	1.86 ± 0.23
C22:6n3	1.40 ± 0.38	1.46 ± 0.16
ΣPUFA^3^	38.18 ± 3.47	37.51 ± 1.66
Σn-3 PUFA^4^	8.74 ± 2.18	10.83 ± 1.37
Σn-6 PUFA^5^	29.45 ± 1.82	26.68 ± 1.95*
n-3/n-6 PUFA	0.30 ± 0.07	0.41 ± 0.07*

* and ** indicate significant differences between the SBO and DGO groups at P < 0.05 and P < 0.01, respectively.

*^1^ΣSFA: saturated fatty acids (C14:0, C16:0, C18:0, C20:0).*

*^2^ΣMUFA: monounsaturated fatty acids (C16:1, C18:1n9, C20:1, C22:1n9).*

*^3^ΣPUFA: polyunsaturated fatty acids (C18:2n6, C18:3n3, C18:3n6, C20:2n6, C20:3n3, C20:4n6, C20:4n3, C20:5n3, C22:6n3).*

*^4^Σn-3 PUFA: polyunsaturated fatty acids (C18:3n3, C20:3n3, C20:4n3, C20:5n3, C22:6n3).*

*^5^Σn-6 PUFA: polyunsaturated fatty acids (C18:2n6, C18:3n6, C20:2n6, C20:4n6).*

## Discussion

This study proposed a two-step vacuum-mediated conversion of EE and FFA from SSBO to DAG-enriched oil by catalyzed ANL-MARE. Enzymatic production of DAG from different materials has been reported in recent studies (14, 36, 37). Li et al. obtained 44.8% medium-chain, and medium- and long-chain fatty acid DAG *via* esterification of high purity monoacylglycerols (MAG) and caprylic acid catalyzed by Novozyme 435 (35). FFA could esterified with glycerol or MAG to produce a higher DAG yield *via* esterification reaction compared to the other reactions (27, 38). Nevertheless, high purity of free fatty acids is an expensive feedstock for production of DAG, which is not suitable for the actual application. Additionally, high-acid oil exists in natural sources, such as high-acid rice bran oil with 30 to 40% FFA. Several studies have reported that high-acid rice bran oil was used for production of DAG (about 27.98%-38.99% yield) by catalysis of Lipozyme RM IM, and the process not only was effective to reduce FFA, but also was utilized to produce high-value oils (23, 39). DAG also could be prepared by lipase-catalyzed glycerolysis of TAG oil, in which could obtain about 20 to 60% DAG content (26, 40). Moreover, few studies utilized fatty acid ethyl esters as an acyl donor for production of DAG by enzymatic glycerolysis with glycerol (21, 41). Our findings demonstrated that DAG-enriched oil was produced by combined glycerolysis of EE and esterification of FFA with glycerol, using a low-cost refined SSBO containing a high content of EE and FFA as raw material. A high content of DAG (around 66%) was obtained in this study, especially a higher content of 1,3-DAG (62.62%), which was higher than that of esterification of high-acid oil and glycerolysis of TAG oil in the previous studies (19, 37, 39). These results were attributable to the higher conversion of EE and FFA to DAG through a two-step vacuum-mediated catalysis by 1,3-specific lipase ANL-MARE. Previous studies have reported that a single FFA or EE are transformed into DAG through a single reaction process (21, 37), and FFA, as an acyl donor, has shown a higher reaction rates and conversions than EE (42). However, few studies performed one-pot conversion of the mixture substrate of EE and FFA to DAG by an enzymatic method. The reactions were comprised of glycerolysis and esterification, because EE and FFA could serve as acyl group donors. Our results suggested that the lipases showed different behaviors between glycerolysis of EE and esterification of FFA with glycerol. A high vacuum level favored the esterification of FFA, whereas a low vacuum level was conducive to the glycerolysis of EE. In line with previous studies, most glycerolysis is carried out under micro-aqueous environment, but esterification reaction requires a lower water content (14). Therefore, a two-step vacuum process was proposed for maximizing the conversions of EE and FFA to DAG. In the first step, the reaction was carried out within an earlier stage under a low vacuum level, in which the glycerolysis activity of EE was superior to the esterification activity of FFA. After most of EE were transformed, a high vacuum level was applied to transform the residual FFA in the reaction mixture. After optimized reaction conditions, the DAG yield achieved 66% and the conversion rates of EE and FFA were up to 96% and 93%, respectively. Hence, this study has succeeded in the production of DAG-enriched oil through a two-step vacuum-mediated conversion of EE and FFA from refined SSBO by the catalysis of ANL-MARE. Moreover, to sustainably utilize SSBO for the production of DAG-enriched oil is not only high-value use of SSBO, but also lends great economic potentiality.

Extensive human and animal studies have reported that dietary DAG oil, especially 1,3-DAG, could reduce postprandial TG levels, lower serum lipids, suppress fat accumulation and body weight gain (3–7). The nutritional study of DAG-enriched oil from SSBO was also evaluated in fish. Although DAG-enriched oil had a similar energy value and fatty acid composition to TAG oil (soybean oil), our results suggested that dietary DAG-enriched oil significantly lowered serum levels of TG, TC and LDL-C, hepatic TG and TC levels, and lipid accumulation in body and muscle tissues when compared with TAG oil. Our results were broadly in line with previous research (6, 43). Meanwhile, a recent study has also reported similar results after supplementation of DAG oil rich in 50% commercial 1,3-diolein in Nile tilapia (44). Furthermore, the health benefits of DAG oil rich in 1,3-DAG might be ascribed to the structural differences between DAG and TAG, thereby showing different routes from common TAG oil in absorption and metabolism (10). In the present study, 1,3-DAG was a major constituent of the dietary DAG-enriched oil (up to 62.62% in oil). Based on these findings, it is reasonable for us to postulate that characteristics of 1,3-DAG absorption and metabolism were responsible for the health benefits of dietary DAG-enriched oil in this study. However, future study would be gained insight into the investigation of its molecular mechanism. Meanwhile, dietary DAG-enriched oil could increase protein content in the muscle tissues of fish in the present study. The benefit of DAG in the enhancement of protein absorption and deposition might be attributed to the feature of DAG emulsification. Previous studies have demonstrated that emulsifier positively affected on nutrient digestibility in animals (45). DAG might be able to promote the incorporation of nutrients into micelles and increase the absorption and bioavailability of nutrients. Hence, DAG-enriched oil would be a prospective application in edible oil and lipid-based delivery systems for the enhancement of the absorption and bioavailability of nutrients or nutraceuticals. Finally, dietary DAG-enriched oil also resulted in a lower percentage of n-6 PUFA, a high percentage of C18:3n3, C20:3n3, and total n-3 PUFA, as well as a higher ratio of n-3/n-6 PUFA in this study. These findings were similar to research showing that postprandial palmitic acid, stearic acid, and linoleic acid levels were lower after the consumption of medium-chain DAG (9). Additionally, the hydrolysis, absorption, and utilization efficiency of fatty acids are associated with the location of fatty acids on the glycerol backbone (30). Therefore, we concluded that dietary DAG-enriched oil could improve fatty acid composition in muscle tissues. Together, DAG-enriched oil exhibited beneficial effects on lowering lipids in the serum, liver, muscle tissues, and body of fish and enhanced the absorption and deposition of protein.

## Conclusion

The DAG-enriched oil was successfully prepared by a combined glycerolysis and esterification using SSBO containing a high content of EE and FFA as raw material and ANL-MARE as a catalyst. To maximally transform EE and FFA into high DAG content, a two-step vacuum-mediated conversion was proposed in one-pot enzymatic catalysis. Furthermore, the nutritional properties of DAG-enriched oil in fish were improved compared with TAG oil, in which dietary DAG-enriched oil reduced lipid levels in serum and liver, decreased lipid accumulation, and increased protein content in body and muscle tissues. Thus, this study provided a promising way in the production of DAG-enriched oil from SSBO and expanded its application in nutrition, which was of great significance for the development of DAG and the sustainability of oil industry. Further study is needed to prepare higher purity of DAG and to understand its mechanism of action to lower lipid and increase the bioavailability of nutrients.

## Data Availability Statement

The raw data supporting the conclusions of this article will be made available by the authors, without undue reservation.

## Ethics Statement

The animal study was reviewed and approved by Institutional Animal Care and Use Committee of the South China Agricultural University.

## Author Contributions

KF, WD, YL, and YC designed the experiment. KF, HF, GL, and QK conducted enzymatic synthesis of DAG-enriched oil and the animal experiments. KF, HF, MS, and YJC did experimental analysis and analyzed the data. KF, JF, LW, QH, and YC wrote and revised the manuscript. All authors contributed to the article and approved the submitted version.

## Conflict of Interest

WD is employed by Guangdong Huiertai Biotechnolgy Co. Ltd. JF is employed by Guangdong Meiweixian Flavoring Foods Co. Ltd. The remaining authors declare that the research was conducted in the absence of any commercial or financial relationships that could be construed as a potential conflict of interest.

## Publisher’s Note

All claims expressed in this article are solely those of the authors and do not necessarily represent those of their affiliated organizations, or those of the publisher, the editors and the reviewers. Any product that may be evaluated in this article, or claim that may be made by its manufacturer, is not guaranteed or endorsed by the publisher.
