# Supplementary material for: Enzymatic Synthesis of Diacylglycerol-Enriched Oil by Two-Step Vacuum-Mediated Conversion of Fatty Acid Ethyl Ester and Fatty Acid From Soy Sauce By-Product Oil as Lipid-Lowering Functional Oil
Source: Front Nutr. 2022 Apr 27;9:884829. doi: 10.3389/fnut.2022.884829 (PMC9093691; doi:10.3389/fnut.2022.884829)
Supplement: Supplementary file 1 [file Data_Sheet_1.docx]

**Supplementary Table 1.** Ingredient formulation (%) and proximate composition of the DGO and SBO diet fed to fish.

| Ingredients (%) | Soybean oil group (SBO) | DAG-enriched oil group (DGO) |
| --- | --- | --- |
| Rape seed meal | 24.00 | 24.00 |
| Soybean meal | 38.00 | 38.00 |
| flour | 26.18 | 26.18 |
| Corn starch | 2.87 | 2.87 |
| Soybean oil | 4.30 | - |
| DAG-enriched SSR oil | - | 4.30 |
| Others | 4.65 | 4.65 |
| Total | 100.00 | 100.00 |
| Analyzed proximate composition (%) | | |
| Moisture | 8.38 | 8.23 |
| Crude protein | 36.02 | 35.67 |
| Crude lipid | 4.69 | 4.63 |
| Ash | 6.42 | 6.36 |


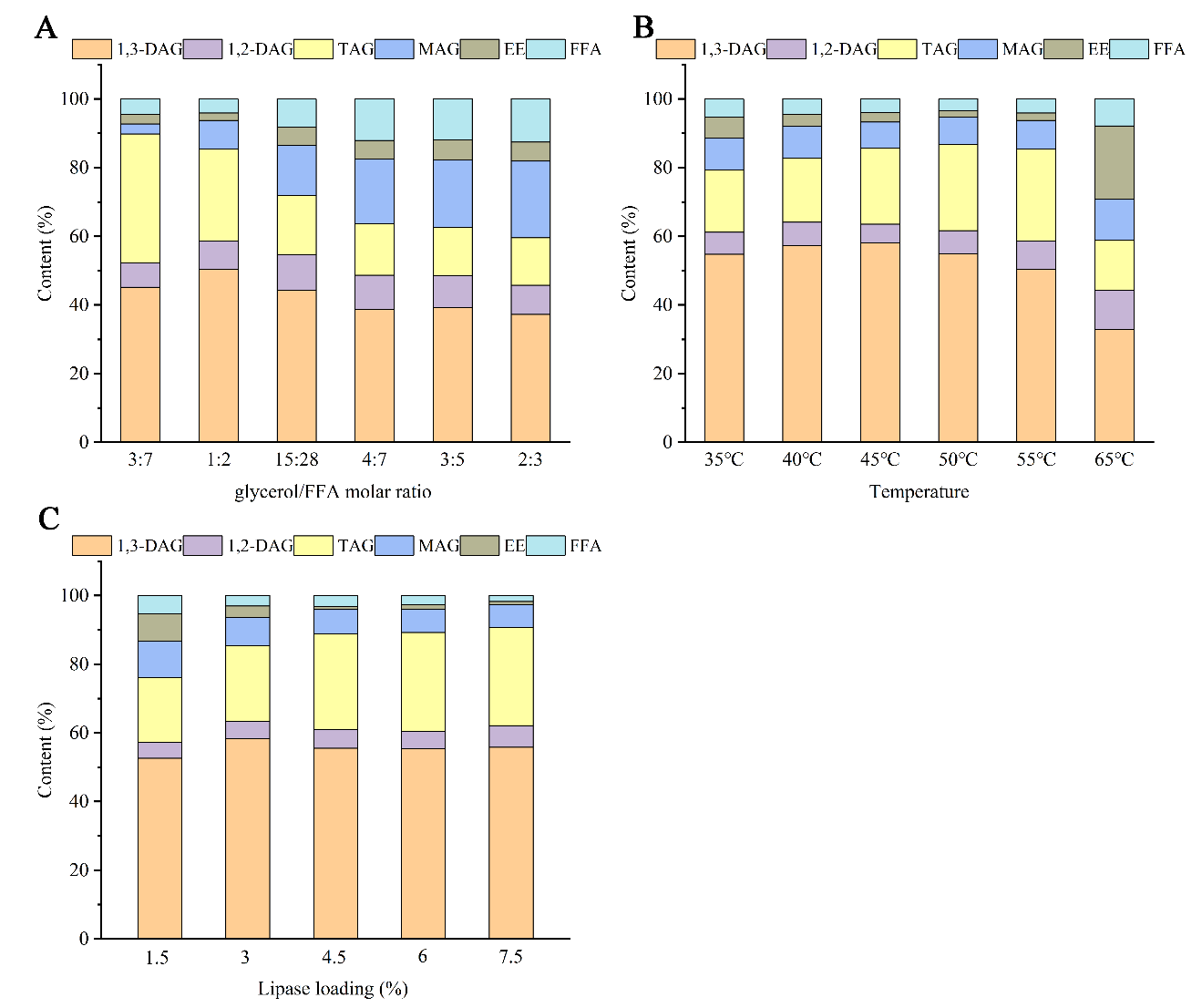


**Supplementary Figure 1.** Effects of substrate molar ratio (glycerol/FFA and EE) (A), temperature (B), ANL-MARE loading (C) on lipid class composition in the crude product.


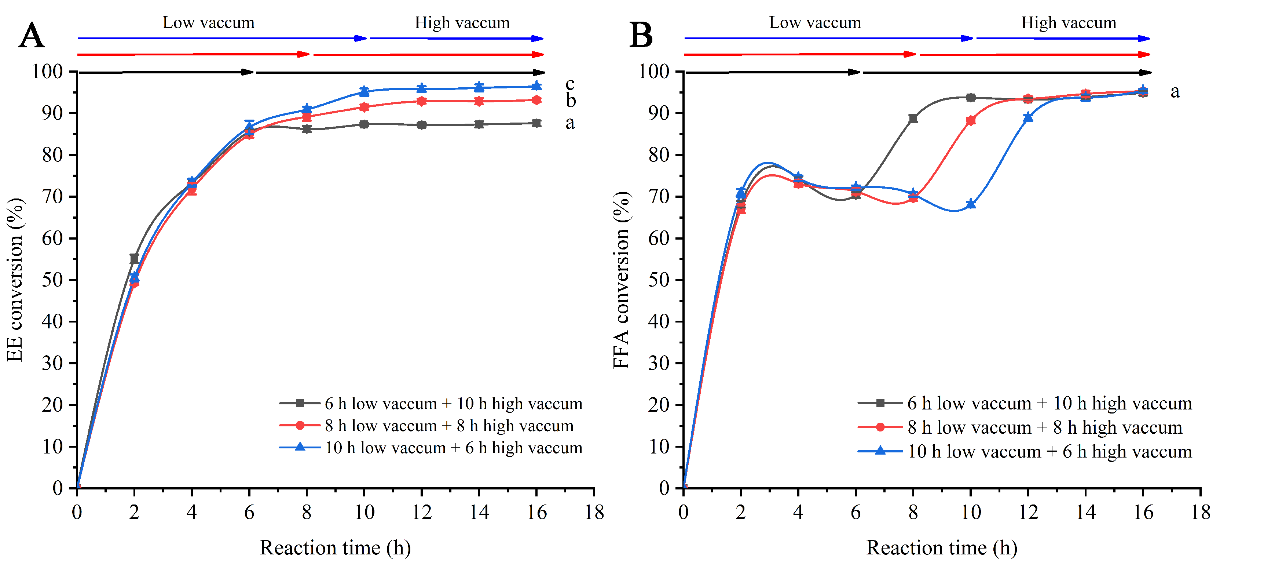


**Supplementary Figure 2.** Effect of reaction time on the conversions of EE (A) and FFA (B).


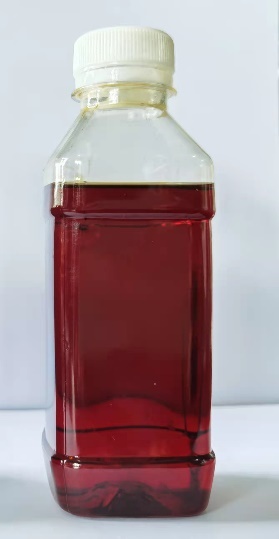


**Supplementary Figure 3.** The sample of DAG-enrich oil after scale-up synthesis from SSBO


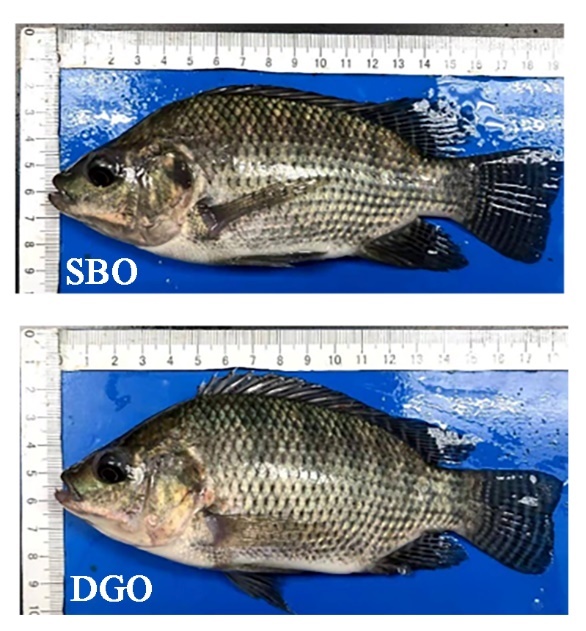


**Supplementary Figure 4.** Representative photographs of fish in the SBO and DGO groups.





**Supplementary Figure 5.** Effects of DAG-enriched oil on serum levels of aspartate aminotransferase (AST) and alanine aminotransferase (ALT) in fish.
